# Fertility Preservation in Female Children and Adolescent Cancer Patients

**DOI:** 10.3390/children12050647

**Published:** 2025-05-16

**Authors:** Min Wang, Chao Yang

**Affiliations:** Department of Surgical Oncology, National Clinical Research Center for Child Health and Disorders, Ministry of Education Key Laboratory of Child Development and Disorders, China International Science and Technology Cooperation Base of Child Development and Critical Disorders, Children’s Hospital of Chongqing Medical University, Chongqing 400014, China; wangmin2506@163.com

**Keywords:** fertility preservation, cancer, pediatric, adolescent, female

## Abstract

The five-year survival rate for childhood cancer now exceeds 80%, leading to an increasing number of young women who may confront infertility in the future due to the gonadotoxic effects of surgery, chemotherapy, and radiation. Despite current guidelines advocating for fertility preservation counseling and necessary reproductive protection measures for all patients, significant barriers and ethical considerations persist, particularly within the pediatric and adolescent female population. In this review, we provide an overview of the impact and mechanisms of anti-tumor therapies on ovarian function, fertility preservation strategies for pediatric and adolescent patients, and the associated costs and ethical considerations that need to be addressed.

## 1. Introduction

Cancer remains one of the paramount causes of mortality among children and adolescents worldwide [1]. Due to remarkable advancements in modern medical technology and the holistic application of diverse cancer treatment modalities, long-term survival rates for cancer patients have substantially increased. Since 1975, the mortality rates for all children, adolescent, and young adult (CAYA) cancers combined have consistently declined by an average of 2.1% annually. Currently, the five-year overall survival rate for childhood cancers has surged to over 80%, according to the SEER (Surveillance, Epidemiology, and End Results) database [2]. As an ever-growing number of pediatric cancer patients achieve long-term survival, enhancing their long-term quality of life becomes progressively important.

Cancer treatment encompasses a variety of modalities, including surgery, radiation therapy, immunotherapy, and chemotherapy, tailored to the type and stage of the disease. Regrettably, some of these therapeutic approaches possess gonadotoxic properties, which have the potential to disrupt folliculogenesis and induce premature ovarian failure (POF) [3]. Endocrine and reproductive complications resulting from ovarian dysfunction are long-term sequelae of significant concern for female CAYA survivors. Although many survivors can conceive, the potential loss of fertility remains a prevalent concern among patients, their parents, and medical caregivers [4]. For female CAYA patients with cancer who have not yet fulfilled their reproductive aspirations, the dual objectives of preserving fertility and maintaining ovarian endocrine function while ensuring effective tumor control pose a significant challenge. Hence, there is a growing demand for the convergence of reproductive medicine and oncology, a burgeoning field known as oncofertility.

Presently, strategies to safeguard ovarian function are formulated based on the mechanisms by which cancer treatments inflict ovarian damage. These strategies include fertility-sparing surgeries, pharmacological interventions to prevent chemotherapy-induced ovarian damage, radiotherapy sparing fields, and ovarian transposition prior to radiation therapy. These methods have demonstrated varying degrees of efficacy in protecting ovarian function. Institutions are increasingly offering oocyte cryopreservation (OC), embryo cryopreservation (EC), and ovarian tissue cryopreservation (OTC), thus expanding the possibilities for restoring endocrine function and fertility in female CAYA cancer survivors.

By summarizing the available literature, this review provides an overview of the impact, mechanisms, and fertility preservation strategies in female CAYA patients. It examines the limitations of current evidence and explores potential directions for advancement, aiming to optimize treatment protocols and safeguard reproductive potential in affected individuals, thereby contributing to enhanced long-term quality of life.

## 2. The Impact of Cancer Treatment on Ovarian Function and Its Underlying Mechanisms

Oocytes in the human ovary are derived from the proliferation and maturation of primordial germ cells. The highest number of oocytes is available around 5 months of pregnancy, followed by a continuous decline after birth. Primordial follicles serve as a resting pool of germ cells from approximately 17 weeks of gestation. Within this pool, follicles are recruited and activated to grow. Only a few follicles will ovulate, while the rest will undergo apoptosis. The number of oocytes in this pool will gradually decrease until none remain, resulting in menopause when the count is around 1000 [5]. The destruction of ovarian tissue, regardless of the cause, causes a decline in the number of follicles and leads to premature exhaustion of the follicle pool, ultimately resulting in POF. Given that the pool of primordial follicles in women is finite, cancer treatments can significantly deplete this oocyte reservoir due to their cytotoxic effects. This depletion may result in a diminished ovarian reserve, infertility, and the onset of premature menopause.

Ovarian dysfunction primarily presents as estrogen deficiency and/or a decline in ovarian reserve, leading to issues such as delayed puberty, menstrual irregularities, and early menopause [6]. It can also shorten the reproductive period and, in severe cases, lead to primary ovarian insufficiency (POI) and infertility [6]. In a cohort studying endocrine dysfunction in medulloblastoma survivors, Stern et al. found a failure of spontaneous initiation of puberty in two of four children and reduced ovarian reserve in all four [7]. Armstrong et al. found that survivors of central nervous system tumors who underwent cancer treatments were more likely to experience the onset of menarche before the age of ten compared to their siblings (11.9% vs. 1.0%). They were also more likely than their siblings to experience the onset of menarche after age 16 (10.6% vs. 1.9%). Both findings were statistically different [8]. The St. Jude Lifetime Cohort Study in 2017 reported that the prevalence of POI in childhood cancer survivors was 10.9% [9].

Patients treated with alkylating agents, stem cell transplantation, abdominal/pelvic radiotherapy, total body irradiation, uterine radiotherapy, and pituitary radiotherapy have an increased risk of infertility [10]. The likelihood of survivors becoming pregnant and having live births is significantly reduced, with the disparity becoming more pronounced after the age of 30. Regardless of exposure to alkylating agents, the chances of pregnancy and live birth are notably lower, and the risk for those with a history of exposure is higher [11].

### 2.1. Surgery

Surgical interventions for tumors affecting the ovaries and adjacent organs can exert both direct and indirect influences on ovarian function. Direct effects primarily encompass radical tumor surgeries that may result in the loss of ovarian tissue, tumor resection leading to tissue excision, and the potential for postoperative adhesions that obstruct the fallopian tubes, coupled with fluid accumulation that can impede fertilization. Indirect effects mainly involve ischemia to the ovaries induced by surgical procedures, which can lead to a reduction in follicular count.

A long-term follow-up study involving 706 pediatric and adolescent cancer survivors revealed that 13.7% of the survivors had entered menopause, with a median age of 44 years, ranging from 18 to 55 years. Notably, 36.0% of the menopausal cases within this cohort were attributed to surgical interventions [12]. Recent research indicates that the resection of a unilateral ovary can result in a 50% reduction in AMH levels in patients [13], potentially impacting the short-term growth and development of children. Should both ovaries be surgically removed, the patient will inevitably experience endocrine dysfunction and infertility.

### 2.2. Chemotherapy

Chemotherapy drugs can influence various stages of follicle development, encompassing the reserve of primordial follicles, their activation, and the subsequent stages of follicle maturation. The principal mechanisms of damage include follicular apoptosis, “burnout” follicles, and impairment of the ovarian stroma and blood vessels [14,15,16].

The extent to which chemotherapy impacts ovarian function is largely contingent upon the types and doses of chemotherapy agents administered, the patient’s age, and the initial levels of AMH. Patients who are diagnosed at an older age and those with initially low AMH levels often exhibit poorer ovarian reserve function. These individuals, who already have compromised ovarian function, are more vulnerable to the deleterious effects of chemotherapy on the ovaries. Conversely, younger patients typically possess more robust ovarian function; they may have a larger follicle pool, enabling residual follicles post-chemotherapy to maintain ovarian function during the early reproductive years.

Chemotherapy agents are categorized into high-risk, medium-risk, and low-risk groups based on their mechanisms of action and gonadotoxic potential. Alkylating agents, such as cyclophosphamide, ifosfamide, and chlorambucil, are classified within the high-risk category [17]. To evaluate the relationship between drug dosage and toxicity, researchers have introduced the concept of the cyclophosphamide equivalent dose (CED) [18]. Studies have shown that doses exceeding 8 g/m^2^ of alkylating agents are associated with ovarian failure [9]. Recent research indicates that a CED greater than 6 g/m^2^ has significant gonadotoxic effects [19]. This finding aligns with recommendations from groups such as the PanCareLIFE guidelines and the International Late Effects of Childhood Cancer Guideline Harmonization Group (IGHG), which propose lowering the threshold for high-risk gonadal treatment from 8 g/m^2^ to 6 g/m^2^.

### 2.3. Radiotherapy

Radiotherapy is widely acknowledged for its detrimental impact on the ovarian reserve. The mechanisms underlying follicular damage induced by radiotherapy encompass the following: (1) the induction of ionization within macromolecular DNA, resulting in multiple disruptions to the DNA double helix structure, which directly precipitates cell apoptosis; (2) the generation of intracellular reactive oxygen species (ROS), which heightens oxidative stress and compromises antioxidant defense mechanisms, thereby indirectly leading to cell apoptosis; and (3) the impairment of ovarian stromal blood vessels, culminating in ischemic necrosis, atrophy, and fibrosis of ovarian tissue.

The gonadotoxic effects of radiotherapy are contingent upon various factors, including the patient’s age, radiation field, total dose, and dose per fraction, with single doses proving more toxic than multiple fractions [20]. Craniospinal irradiation can indirectly influence ovarian function by affecting the hypothalamic–pituitary axis, while scattered doses may also directly impact the ovaries. Research has found that radiotherapy doses exceeding 30 Gy to the hypothalamic–pituitary region constitute a significant risk factor for infertility in pediatric cancer patients. Doses ranging from 30 to 55 Gy are associated with precocious puberty, whereas doses surpassing 55 Gy are more likely to induce gonadotropin deficiency, manifesting as delayed or arrested puberty [21].

Focusing on the specific impacts of radiotherapy, it is well established that half of the total number of ovarian follicles are lost at doses as low as 2 Gy (lethal dose 50%), which is significantly lower than the doses administered in a curative setting [22]. After whole pelvic radiation (20–30 Gy), ovarian failure rates can reach as high as 97% [23]. The tolerance of the ovaries to radiation therapy gradually decreases with age. Van Dorp developed a mathematical model based on data regarding the rate of oocyte decline, suggesting that the effective sterilizing dose is 20.3 Gy in infants, 18.4 Gy at age 10, and 16.5 Gy at age 20 [24]. However, it is crucial to note that while younger patients exhibit higher ovarian tolerance to radiotherapy, the inverse is true for the effects on the uterus. The younger the patient at the time of radiation, the more pronounced the adverse effects on the uterus, particularly when radiation is administered to prepubertal girls [25]. This may result in an inability to conceive normally in adulthood, even if ovarian function remains sufficient to meet reproductive needs. Chiarelli et al. evaluated pediatric cancer survivors (*n* = 340) and found an increased risk of preterm birth and low-birth-weight infants at pelvic and/or abdominal doses >2.5 Gy (OR 3.49, 95% CI 1.26–9.72) [26].

### 2.4. Immunotherapy

Immunotherapies, including immune checkpoint inhibitors, immunomodulators, monoclonal antibodies, and CAR-T, can increase levels of proinflammatory cytokines and immune-related adverse events, potentially exacerbating fertility issues. Inflammation related to immunotherapy, characterized by cytokine imbalances and the activation of pathways such as AMPK/mTOR, has been identified as a contributing factor in the mechanisms of fertility impairment [27]. However, there is a lack of research data regarding the impact of immunotherapy on fertility in pediatric and adolescent oncology patients, as the application of immunotherapy in this population is still in the clinical trial phase [28]. The absence of fertility rate data highlights the need for enhanced research efforts in this area.

### 2.5. Recovery of Ovarian Function After Cancer Treatment

Regular monitoring of ovarian reserve is essential after cancer treatments. The resumption of normal pubertal development and the restoration of menstrual cycles following cancer treatments suggest a partial recovery of ovarian endocrine function. Alf et al. found that approximately half (7/15) of female children with impaired ovarian function due to anti-tumor therapy exhibited normal AMH levels 1 year after the end of tumor treatment [29]. However, research indicates that pubertal development alone cannot serve as a reliable predictor of gonadal function in early adulthood [30]. Even among CAYA cancer survivors who experience normal or restored menstrual cycles, the AFC may be diminished, signaling a reduced ovarian reserve [31]. Thus, it can be inferred that the normalization of pubertal development and menstrual cycle restoration does not necessarily correlate with the preservation of ovarian reserve post-puberty. Therefore, ongoing monitoring of ovarian function in female pediatric cancer survivors is essential to ensure that any latent or progressive decline in reproductive function is promptly identified and managed.

## 3. Fertility Preservation in Cancer Treatments

In addressing ovarian function damage induced by oncological therapies, the guiding principle should emphasize prevention over treatment. From a fertility preservation perspective, the current primary strategies include EC, OC, and OTC. However, each technique presents inherent limitations that constrain its broader clinical application. For female CAYA cancer patients, fertility preservation options remain notably restricted, especially for prepubertal patients. Consequently, it is imperative to explore innovative therapeutic avenues that maintain the efficacy of cancer treatments while minimizing ovarian function impairment. This dual focus on effective oncological control and fertility preservation necessitates ongoing research and the development of novel treatment modalities that can offer a balanced approach to patient care.

### 3.1. Ovarian Protection Related to Surgery

Fertility-sparing surgery (FSS), which entails the preservation of the uterus and one or both ovaries or ovarian tissue, has been a subject of debate since the mid-1980s, particularly for young women with early-stage ovarian tumors. Despite the controversy, there is a paucity of long-term follow-up studies evaluating the outcomes of such procedures. However, emerging research indicates that FSS can be a viable option for young women with early-stage ovarian tumors who wish to maintain their reproductive potential. This approach does not seem to negatively impact tumor prognosis, pregnancy outcomes, or neonatal health [32]. Yoo et al. found that patients with premenstrual and pubertal malignant germ cell tumors of the ovary had a high survival rate after fertility-preserving surgery and adjuvant chemotherapy (25/25) and that the majority of these patients experienced normal menarche (8/9) and menses (15/16) [33]. A comprehensive review published in 2018 systematically examined ovarian preservation strategies for the treatment of pediatric gynecological malignancies [34]. The findings robustly support the safety and efficacy of fertility-sparing surgery and ovarian conservation in managing ovarian cancer and genitourinary rhabdomyosarcoma in pediatric and adolescent patients. Furthermore, the COG advocates for the preservation of the ipsilateral fallopian tube in cases where it is not involved in malignant germ cell tumors, underscoring a growing consensus on the feasibility and safety of fertility-preserving approaches in these specific oncological contexts [35].

### 3.2. Current and Emerging Strategies for Ovarian Protection in Chemotherapy

In addition to minimizing the use of gonadotoxic chemotherapeutic agents, the employment of ovarian suppressive and neuroprotective agents has shown promise in mitigating the deleterious effects of chemotherapy on ovarian function. In conventional chemotherapy regimens, cytotoxicity-associated ovarian insufficiency involves the depletion of the PF pool through apoptotic or hyperactivation mechanisms, particularly mediated through the ABL/TAp 63 and PI 3 K/Akt/mTOR pathways. According to an in vivo study using cyclophosphamide-treated mice, AS101 reduced the cyclophosphamide-induced activation of ovarian PTEN/PI3K/Akt pathway proteins, reduced follicle loss, and increased AMH concentrations [36]. Sonigo et al. demonstrated that AMH can inhibit PF activation by down-regulating FOXO 3A phosphorylation levels in a cyclophosphamide-treated pubertal mouse model, leading to a reduction in PF depletion [37]. In addition, in animal models, mTOR stimulators increase primordial follicle activation, whereas mTOR inhibitors block the transition from primordial to primary follicles. Using the inhibition of mTOR complex 1 (mTORC 1) with the clinically approved drug everolimus (RAD 001) or mTORC 1/2 with the experimental drug INK 128 in a clinically relevant mouse model of cyclophosphamide-induced gonadotoxicity, Goldman et al. demonstrated that mTOR inhibition preserves ovarian reserve, primordial follicle counts, serum anti-mullerian tube hormone levels, and fertility [38]. Granulocyte colony-stimulating factor (G-CSF) was investigated as a fertility-protective agent against chemotherapy-induced vascular injury [39].

Although there is still a lack of clinical studies directly applying these methods, ongoing and deeper exploration of the mechanisms involved, along with continued research into the development and application of molecules that limit ovarian damage from chemotherapy, is necessary, as it is expected to reduce the need for invasive fertility preservation techniques.

### 3.3. Ovarian Protection During Radiation Therapy

By selecting the appropriate beam energy and radiotherapy plans, it is possible to minimize the radiation dose to the ovaries without compromising cancer treatment effectiveness. Kelsey et al. [40] developed a new algorithm based on ovarian LD50 and age-related follicle number decline specificity that calculates the Effective Sterilizing Dose (ESD). This algorithm helps patients choose the optimal radiotherapy regimen and predicts the age of ovarian insufficiency following radiotherapy, serving as a reference for their fertility counseling. Fractionated radiotherapy can help preserve ovarian function by administering lower and increasingly fractionated doses, improving the potential for repairing the damaged follicular population [41]. Advanced targeted radiation techniques are used to maximize target coverage while employing highly conformal dose distributions to protect normal organs. For patients with lymphoma receiving abdominal and pelvic radiotherapy, involved-site radiation therapy (ISRT) significantly reduces ovarian radiation doses and extends the time from treatment to premature menopause [42].

Ovarian transposition (OT) was initially proposed as a procedure to preserve ovarian function in children with cancer undergoing abdominal and pelvic radiotherapy. Guidelines recommend this procedure for adolescents and young adults who require pelvic radiotherapy [43,44,45]. Research has identified optimal distances between the ovaries and the planned target volume, with distances greater than 3.3 cm resulting in ovarian radiation doses below 4 Gy, and distances greater than 2.4 cm achieving doses below 5 Gy [46]. The preservation of ovarian function after OT varies significantly, with preservation rates ranging from 32% to 88% [47,48]. This variability may be due to changes in the spatial position of the ovaries, influenced by factors such as bladder fullness, which could cause the ovaries to move back into the radiation field during treatment. The location of the transposed ovary is closely associated with ovarian survival, and the distance between the edge of the radiation field and the transposed ovaries affects the successful preservation of ovarian function. Transposing the ovaries more than 1.5 cm above the iliac crest can significantly reduce the damage caused by pelvic radiotherapy [49,50].

Furthermore, new radiotherapy technologies and equipment can reduce radiation damage to the ovaries. Proton beam therapy is an effective treatment method for pediatric and adolescent tumors, with the potential to completely avoid radiation exposure when the ovaries are located in deeper regions. However, this technology is not yet widely available [51]. More findings have emerged from animal experiments aimed at reducing ovarian damage from radiotherapy. For example, Said et al. found that sodium selenite (SS) significantly ameliorated radiation-induced ovarian and uterine oxidative stress by decreasing lipid peroxide levels and increasing both glutathione levels and glutathione peroxidase activity in a rat model of radiological ovarian injury through long-term treatment with SS [52]. Similarly, Demyashkin et al. found that leukocyte-poor platelet-rich plasma components induced endogenous antioxidant protection, repaired post-irradiation follicular damage, and slowed the progression of radiation-induced premature ovarian failure after electron irradiation [53]. In addition, a study showed that transcutaneous electrical acupoint stimulation (TEAS) can attenuate radiation-induced ovarian failure by inhibiting primordial follicle loss, increasing serum AMH secretion, and inducing antioxidant and anti-apoptotic systems [54].

### 3.4. Fertility Preservation in Cancer Treatment

The National Comprehensive Cancer Network (NCCN) in the United States [55], the European Society for Medical Oncology (ESMO) [56], the International Federation of Gynecology and Obstetrics (FIGO) [57], the European Society of Human Reproduction and Embryology (ESHRE) [43], and the American Society of Clinical Oncology (ASCO) [58] have issued guidelines for fertility preservation in children and adolescents. These guidelines recommend options such as EC, OC, and OTC for fertility preservation in this population. It is strongly recommended that fertility preservation counseling be offered to all pediatric and adolescent cancer patients who will undergo alkylating agent chemotherapy, ovarian or cranial radiation, stem cell transplantation, or unilateral oophorectomy. For patients who have received doses of alkylating agents greater than 6–8 g/m^2^, ovarian radiation, or stem cell transplantation after puberty, OC or EC is strongly recommended, whereas for prepubertal patients, OTC is moderately recommended. For other cancer control treatments, OC or EC is moderately recommended only for post-pubertal patients [44]. A summary of preservation options based on the timing of anticancer therapy is provided in Table 1.

EC and OC are the standard methods of fertility preservation for post-pubertal females. While EC is a well-established technique, it requires sperm and is limited by this requirement. OC involves stimulating a patient’s ovaries, collecting oocytes, and freezing them to implant once the patient has recovered from cancer. Furthermore, OC can be performed without a partner, making it the preferred choice for most post-pubertal females. Both EC and OC involve ovarian stimulation with gonadotropins and subsequent oocyte retrieval, which may cause a delay in chemotherapy [59]. GnRH antagonists offer faster suppression of ovarian function without the need to wait for 14 days, which can be advantageous for certain cancer patients. However, there is limited data available on the use of GnRH antagonists alone for this purpose. If a patient’s cancer treatment cannot be delayed by 14 days, OTC could be an option [60].

For most prepubertal cancer patients, OTC is currently considered the primary and often the only option for fertility preservation. OTC involves removing ovarian tissue and cryopreserving it for potential future transplantation. The primary goal of this approach is to preserve the opportunity to restore ovarian function in the future. Notably, OTC not only restores fertility but also reinstates endocrine function and initiates puberty, which is currently considered its primary objective. In 2019, the American Society for Reproductive Medicine (ASRM) stated that OTC and transplantation are no longer considered experimental techniques [61]. Studies have reported a high recovery rate of ovarian endocrine function, reaching up to 95% following transplantation [62]. Poirot et al. reported that a 13-year-old girl with no signs of puberty, who underwent OTC prior to allogeneic hemopoietic stem-cell transplantation, later received autologous ovarian tissue transplantation (OTT), which successfully induced puberty [63]. At the time of the study, she had attained normal height, developed secondary sexual characteristics, and begun menstruating. A multicenter study revealed that pregnancy and live birth rates among CAYA cancer survivors who underwent OTC were 50% and 41%, respectively [64]. The largest series to date included 285 females who had their cryopreserved ovarian tissue thawed and transplanted with the intent of conceiving, yielding pregnancy and live birth rates of 38% and 26%, respectively [65]. To date, over 200 live births globally have been attributed to this technique [66]. Notably, this includes two cases where the tissue was frozen before menarche (one prepubertal and one during puberty), demonstrating potential for future fertility preservation in prepubertal patients.

Nevertheless, the safety and efficacy of these procedures require further validation through larger, comprehensive studies [67,68]. Based on the literature on fertility preservation in female children with cancer between 2004 and 2022, it was found that 501 patients underwent OTC, of whom 5.9% (30/501) proceeded to ovarian tissue transplantation (OTT) [69]. Of these, 27 patients desired pregnancy, resulting in a pregnancy rate of 33% (9/27) and a live birth rate of 67% (6/9). The extraction of ovarian tissue before cancer treatment raises several concerns, including (1) the risk of delaying anticancer therapy; (2) the surgical risks associated with laparoscopic or open surgery used to obtain ovarian tissue; and (3) the possibility of reintroducing malignant cells [70,71]. Lotz et al. found no surgical complications and no delays in cancer therapy among 52 pediatric oncology patients who underwent laparoscopic or open surgery to obtain ovarian tissue (mean age: 14.8 ± 2.3 years; range: 6–17 years) [72]. Kasei et al. reported that in three children with tumors under the age of 3, after successful laparoscopic salpingo-oophorectomy was performed to obtain ovarian tissue for fertility preservation prior to cancer therapy, no surgical complications such as bleeding or infection were observed [73]. There is concern about the potential presence of tumor cells in the tissue, particularly in cases of leukemia and lymphoma. Previous reports have identified tumor cells in ovarian tissue cryopreserved from patients with Hodgkin lymphoma [74]. Recently, a study involving 183 patients who underwent OTC found malignant tumors in four patients (2.2%) [75]. Dolmans et al. described a case where a 9-year-old girl’s tumor recurred after OTT; however, this recurrence was attributed to the underlying disease rather than the transplantation itself, as the site of recurrence was distant from the graft site and located near the primary tumor location [65]. They further suggested that, to reduce the risk of reintroducing malignant cells, ovarian tissue should be harvested when the patient is in complete remission. Prior studies have shown that chemotherapy administered before OTC does not significantly affect graft follicle density or reproductive outcomes [76,77,78]. To address the risk of reintroducing malignant cells, Grubliauskaite et al. reviewed the available literature and found that this risk could be minimized by detecting microinvasive disease (MID) in the ovarian tissue prior to transplantation [79]. Meanwhile, Moghassemi et al. demonstrated that OR141-loaded nanovesicles could effectively eradicate leukemic cells from ovarian tissue [80]. Nevertheless, the safety of ovarian tissue transplantation in cancer survivors is an area that still requires further investigation.

### 3.5. Emerging Strategies for Fertility Preservation

In recent years, the application of stem cells in treating various degenerative and injury-related diseases has garnered significant attention due to their multipotent differentiation potential. This promising avenue offers novel directions for the clinical management of POI [81]. Preclinical studies have investigated the use of mesenchymal stem cells (MSCs), embryonic stem cells, induced pluripotent stem cells, and germ cells to address ovarian dysfunction [82,83]. MSCs, in particular, show great promise in improving ovarian function following cancer treatment, demonstrating beneficial therapeutic effects across various animal models of tumor treatment-related ovarian dysfunction [84].

Artificial ovaries represent another groundbreaking advancement, capable of mitigating the risk of tumor cell implantation while restoring ovarian endocrine function. This technology holds particular promise for prepubertal cancer patients, for whom traditional fertility preservation methods pose significant challenges. Successful trials in murine models have laid the groundwork for this innovative approach [85], although human trials remain imperative to confirm its efficacy and safety.

Furthermore, as our understanding of chemotherapy and radiotherapy-induced genomic damage to ovarian function deepens, targeting and modifying relevant gene pathways could lead to new therapeutic approaches for reproductive protection. Integrating precision medicine into cancer treatment, along with proactive screening for ovarian damage risk, could usher in personalized fertility preservation strategies. This tailored approach would enhance the efficacy of interventions and align them with the genetic and clinical profiles of individual patients.

## 4. Additional Considerations

Despite the increasing application of fertility preservation techniques in clinical settings, significant gaps remain in pediatric oncology. Fertility preservation for pediatric cancer patients faces ethical, legal, social, and policy issues, including fertility preservation counseling and advice, the unique decision-making concerns associated with pediatric oncology fertility, as well as cultural and cost issues, among others [86].

Current research data are unable to distinguish whether the benefits of fertility preservation outweigh the drawbacks for pediatric and adolescent oncology patients, due to the challenges in assessing the gonadotoxicity of personalized cancer treatments and the limited implementation of fertility preservation measures [87]. Limited studies have been conducted, with short follow-up periods making it challenging to assess long-term reproductive function and potentially underestimating ovarian insufficiency. Comprehensive long-term follow-up is essential to monitor endocrine and reproductive outcomes over time. The diverse and individualized nature of cancer treatments complicates the assessment of gonadotoxicity for specific drugs or methods. Additionally, there is a lack of awareness among parents and healthcare professionals about fertility preservation, leading to inadequate communication with patients and families. Concerns about delaying primary cancer treatment for fertility preservation also hinder the implementation of these strategies.

Decision-making on fertility preservation is also significant, particularly when patients are minors. Differences between the preferences of parents and children regarding fertility preservation can complicate decision-making. To minimize disagreements in fertility preservation decisions, it is crucial to respect minors’ rights and interests while maximizing their future opportunities. Efforts should be made to coordinate and balance the autonomy of both parents and children to optimize the benefits of fertility preservation for minors. Furthermore, the costs of fertility preservation, often not covered by insurance, impose a substantial financial burden on families already facing high cancer treatment expenses, thus limiting their options.

Therefore, evidence-based decision aids (DAs) may serve as valuable tools to support pediatric patients and their families in making informed decisions regarding fertility preservation. The establishment of specialized multidisciplinary fertility preservation teams, together with improvements to relevant legal and regulatory frameworks, can strengthen the delivery of integrated support services for children and adolescents confronting cancer-related fertility challenges [88].

## 5. Conclusions and Future Perspectives

Cancer treatments can affect ovarian function in children and adolescents to varying degrees through different mechanisms of action. The increasing number of cancer treatments with favorable outcomes has justified the emergence of oncofertility programs aimed at mitigating devastating gonadotoxic risks and providing various options for preserving fertility. For the past two decades, many organizations have issued guidelines and recommendations regarding fertility preservation for cancer patients.

For prepubertal patients, some alternative options remain experimental, but the opportunity to offer hope for future generations is invaluable. With the significant improvement in survival rates among CAYA cancer patients, the goals of cancer treatment should shift from solely improving long-term survival to also ensuring reproductive quality.

Therefore, future efforts to deepen our understanding of fertility preservation after cancer treatment will require more basic experimental research and sufficiently long-term prospective clinical studies. These studies should explore the mechanisms of ovarian damage and develop more gonadotoxic treatment options to protect reproductive function. This will significantly increase the number of patients who can benefit from fertility preservation technologies.

## Figures and Tables

**Table 1:** Fertility preservation options for female patients.

	Oocyte Cryopreservation	Embryo Cryopreservation	Ovarian Tissue Cryopreservation
Which patients	Post-pubertal	Post-pubertal	Prepubertal AND Post-pubertal
Impact on cancer treatment	Delays the start time of treatment	Delays the start time of treatment	Does not delay therapy
Rates of birth per embryo transfer	~50%	~56%	25–30% after transplantation
Time needed	2 weeks	2 weeks or more	1–3 days
Limitation	Delays in therapy	Need sperm (almost impossible for children and adolescents)	Potential of reseeding malignant cells
		Religious objections in some areas	Ethical considerations
			Another surgery is needed
